# The effectiveness of police custody assessments in identifying suspects with intellectual disabilities and attention deficit hyperactivity disorder

**DOI:** 10.1186/1741-7015-11-248

**Published:** 2013-11-21

**Authors:** Susan Young, Emily J Goodwin, Ottilie Sedgwick, Gisli H Gudjonsson

**Affiliations:** 1 King’s College London, Institute of Psychiatry, London, UK; 2 Broadmoor Hospital, Crowthorne, UK; 3 Department of Forensic and Neurodevelopmental Sciences, PO23, Institute of Psychiatry, De Crespigny Park, London SE5 8AF, UK

**Keywords:** Intellectual disabilities, ADHD, Conduct disorder, IQ, Risk assessment, Police

## Abstract

**Background:**

Intellectual Disabilities (ID) and Attention Deficit Hyperactivity Disorder (ADHD) are recognized psychological vulnerabilities in police interviews and court proceedings in England and Wales. The aims of this study were to investigate: (a) the prevalence of ID and/or ADHD among suspects detained at a large London metropolitan police station and their relationship with conduct disorder (CD), (b) the impact of their condition on police staff resources, (c) the effectiveness of current custody assessment tools in identifying psychological vulnerabilities, and (d) the use of ‘Appropriate Adults’ in interviews.

**Method:**

A total of 200 individuals in a police custody suite were interviewed and screened for ID, ADHD (current symptoms) and CD.

**Results:**

The screening rates for these three disorders were 6.7%, 23.5% and 76.3%, respectively. ADHD contributed significantly to increased requests being made of staff after controlling for CD and duration of time in custody. This is a novel finding. Reading and writing difficulties and mental health problems were often identified from the custody risk assessment tools, but they were not used effectively to inform on the need for the use of an Appropriate Adult. The frequency with which Appropriate Adults were provided to support detainees in police interviews (4.2%) remains almost identical to that found in a similar study conducted 20 years previously.

**Conclusions:**

The current findings suggest that in spite of reforms recently made in custodial settings, procedures may not have had the anticipated impact of improving safeguards for vulnerable suspects. Detainees with ID and ADHD require an Appropriate Adult during police interviews and other formal custody procedures, which they commonly do not currently receive. The findings of the current study suggest this may be due, in large part, to the ineffective use of risk-assessment tools and healthcare professionals, which represent missed opportunities to identify such vulnerabilities.

## Background

The widely publicized Bradley report [1] raised concerns about the inadequacy of the current provision of health services within the criminal justice system (CJS) in England and Wales. In response, a national Health and Criminal Justice Programme Board was set up, bringing together government departments for health, social care and criminal justice. The Board devised a National Delivery Plan committed to improving the management of offenders with mental health problems, intellectual disability and personality disorder. The subsequent reforms led to considerable change in police services, with changes in screening for vulnerabilities and mental health facilities, and, importantly, healthcare provision and liaison by clinical practitioners (that is, doctors and nurses) who can access NHS records. Following implementation, the success of these changes has yet to be evaluated.

### Vulnerabilities in police detainees with intellectual disabilities

Concerns about the identification of vulnerabilities of suspects in police custody, and suggested amendments to provisions for these, were raised in the early 1990s in studies for the Royal Commission on Criminal Justice [2,3]. This work investigated vulnerabilities in police detainees and identified high rates of individuals in the CJS with unrecognized intellectual disability (ID) or mental health problems [2]. The authors reported that ID was an important vulnerability for 8.6% of the 163 police detainees included in the study. However, there is large variability in reported rates of intellectual disability among persons in police custody. For example, the Bradley review [1] reported on findings from studies suggesting that the prevalence of intellectual disabilities in these settings ranged from 0.5% to 9% of detainees. Variation in the results of the different studies could be due to methods of assessing intellectual disabilities, as practical constraints mean that it is not common to use full diagnostic criteria in research (that is, IQ below 70, significant impairments of adaptive behaviors, and childhood onset). For example, the study by Gudjonsson *et al*. [2] relied on an assessment of intellectual functioning alone, and studies estimating prevalence in prison and probation services have relied on screening measures (for example, [4]). These studies therefore provide estimates rather than precise rates of diagnosis, which is a limitation [5,6].

A broader evaluation of difficulties by the Royal Commission found that 35% of detainees had “problems which might interfere with their functioning or coping ability during police interviewing” ([2], page 15). Hence, the under-identification of vulnerabilities may have serious consequences in terms of impaired capacity to understand their legal rights and cope effectively with police questioning in custody [3,7-11].

For almost 30 years, the Police and Criminal Evidence Act (PACE) [12] and its accompanying Codes of Practice have recommended that vulnerable individuals are provided with special legal protection. This includes the presence of an ‘Appropriate Adult’ during police interviews and a warning to the jury at trial “that there is a special need for caution before convicting the accused in reliance on the confession” (see [13], page 259). The role of an ‘Appropriate Adult’ is to provide support and advice to people with ID and other mental health difficulties [14], and is the main protection for juveniles (detainees aged under 17) and ‘mentally vulnerable’ detainees during interviews by police. However, such provisions can only be implemented when vulnerabilities have been recognized [1-3,15]. To facilitate this, a Royal Commission study [3] recommended a self-identification process whereby detainees participate in a risk assessment screen. Unfortunately, this did not appear to have been successful for ‘mentally vulnerable’ detainees due to continued failure of identification and when vulnerabilities were identified, their needs for an Appropriate Adult were often not appropriately acted upon [14]. The importance of early identification of relevant and pertinent vulnerabilities in the interview process is to ensure fairness and justice [7].

### Vulnerabilities in police detainees with attention deficit hyperactivity disorder (ADHD)

The focus of the previous consultation reports [2,3,15] has largely centered on the needs of offenders with intellectual disabilities (ID). However, the PACE Codes of Practice have been regularly updated and the criteria for psychological vulnerabilities of detainees broadened beyond ID to include “mentally disordered or otherwise mentally vulnerable detained person” ([16], Code C, page 61). The term ‘mentally vulnerable’ refers to “psychological characteristics or mental state which render a witness prone, in certain circumstances, to providing information which is inaccurate, unreliable or misleading” ([7], page 166). It “applies to any detainee who, because of their mental state or capacity, may not understand the significance of what is said, of questions or of their replies” ([16], Section 1G, page 5). Relevant vulnerabilities in a given case may include low intelligence, inability to focus on or cope with the questioning, and high suggestibility or compliance [13]. This includes people with ADHD who present with symptoms of inattention, hyperactivity, impulsivity [17]; executive function deficits [18-21]; disorganization in their behavior and personality [22]; and functional deficits in educational, social and occupational domains [23]. In addition, these individuals tend to give a disproportionate number of ‘don’t know’ responses when questioned, which may be misconstrued as being uncooperative [11], and are prone to make false confessions during police questioning [24,25]. Once in a custodial environment, offenders with ADHD can present a management problem as their symptoms are reportedly associated with aggressive behaviors [26,27], most likely due to their emotional lability and behavioral disinhibition [28]. This may be similarly relevant to police custodial settings where impatience and restlessness may be expressed as requests being made of staff.

Compared with a general population rate of 5.9 to 7.1% for childhood ADHD [29] and 2.5% for adult ADHD [30], international studies have consistently reported disproportionately higher rates of ADHD in offenders (for example, [26,27,31-35]). A 30-year follow-up study conducted in the USA reported that based on a review of arrest records boys identified as hyperactive were significantly more likely to have been arrested, incarcerated or convicted in adulthood, compared with age-matched controls [36]. However, the relationship between ADHD and criminality may be distorted by its association with comorbid conduct disorder [25]. Indeed, in one study, the majority of the sample had conduct problems, making it difficult to attribute a causal role to ADHD [36].

There is conflicting evidence from research as to the influence of each disorder on forensic outcomes. A follow-up study of childhood psychiatric inpatients (n = 541) reported that those with childhood Conduct Disorder (CD) or CD with combined hyperkinetic disorder were at significantly greater risk for adult delinquency, whereas those without CD were no more likely to commit future crime than those with any other psychiatric disorder [37]. In contrast, it has been found that ADHD significantly predicted arrest, conviction or incarceration in adulthood, even when controlling for comorbid CD [38]. Given this discrepancy in the literature, it seems prudent to control for CD when appropriate, particularly as ADHD and CD are known to co-occur at a higher rate than chance [39]. It should also be noted that there is an overlap reported between ADHD and ID (for example, [19]). People with both disorders are likely to have ‘double deficit’ in their cognitive and adaptive functioning, which may lead to greater vulnerability in police interviews and in their capacity to cope more generally within the CJS [19]. Furthermore, a relationship has been reported among all three conditions, with evidence that, among children, those with combined ADHD and ID exhibit greater conduct problems than those with each condition in isolation [40].

### The Bradley Report recommendations

PACE and its accompanying Codes of Practice [12] do not appear to have been successful in addressing police practices for vulnerable detainees. Nearly 20 years since the Royal Commission on Criminal Justice Report [2], the Bradley Report [1], based on nationwide consultations with organizations at all stages of the offender pathway, suggested that the needs of offenders with ID, and those with mental health problems, often remain unrecognized and undetected within the CJS. The Bradley Report recommended improved screening of vulnerabilities and mental health facilities (that is, through the presence of healthcare practitioners (HCPs) and access to NHS medical records) at police stations, which are currently being implemented [16].

The updated Code C of the PACE Codes of Practice [16] stipulated that the custody officer (or other custody staff) has to determine whether a detainee requires medical treatment, an Appropriate Adult, assistance to check documentation and/or an interpreter (Section 35). In addition, “Chief Officers should ensure that arrangements for proper and effective risk assessments must follow a structured process which clearly defines the categories of risk to be considered and the results must be incorporated in the detainee’s Custody Record” (Sections 3.7 and 3.8, page 8). This responsibility involves implementing a response to a specific risk assessment (for example, to assess risk of self-harm and harm to others) and, if this risk is deemed present, increasing levels of monitoring and observation, and calling a suitably qualified HCP (Section 3.9). In turn, this has led to the development of a new Custody Record risk assessment screening tool (with information being derived from a variety of sources, including records, observation, informants and self-report). At the present time this is being incorporated into national police practice and consists of a formal document within the Custody Record that provides (a) the custody officer’s risk assessment and (b) the detainee’s self-reported risk assessment [41]. The focus is both on the needs of the detainee and safety issues surrounding his or her detention. With this in mind, the main aim of the current study was to investigate the effectiveness of this new assessment procedure in identifying psychologically vulnerable detainees with ID and ADHD, and its impact on the use of ‘Appropriate Adults’.

The study was conducted with a group of individuals who were arrested and detained in a police custody suite in South London, where the Bradley recommendations were being implemented, in order to address the following hypotheses. (H1) rates of ID and ADHD in people presenting in police custody will be higher than reported for the general population. (H2) significant comorbidity will be found among ID, ADHD and CD [39,40]. (H3) those with a higher score on screening measures of symptoms of ID, ADHD and CD will make more requests for staff time. (H4) those detainees currently screened as symptomatic for ADHD will consume staff resources over and above CD and ID (that is, the ADHD scores will significantly add to the variance in requests after controlling for the effects of CD and ID).

In addition to the above hypotheses, exploratory analysis was conducted to investigate whether the police Custody Record risk assessment screening tool and/or HCP contact in police custody, successfully identified vulnerable individuals leading to an increased use of Appropriate Adults during police interviews.

## Methods

### Participants

The sample comprised 200 individuals in custody at a police station in South East London. The mean age of the group was 27.5 years (SD = 10.5, range = 16 to 69), most being male (N = 185; 92.5%). Ethnic background was taken from the Custody Record for 194 participants, of whom 95 were classified as white (49.0%), 85 black (43.8%), 13 mixed race (6.7%) and 1 Asian (0.5%). The participants were in custody for a range of alleged offenses (information available for 191 participants) including property (n = 82, 43%), violence (n = 48, 25%), criminal damage (n = 17, 9%), public disturbance (n = 12, 6%), drug-related (n = 8, 4%), driving (n = 5, 3%), sexual (n = 2, 1%) and other offenses (n = 17, 9%), the ‘other’ category mainly included a warrant or breach of court restrictions/bail. All individuals were assessed by custody staff during a routine intake procedure for capacity to participate in the study. This included an evaluation of alcohol and drug intoxication or withdrawal, serious mental health problems, severe communication/comprehension problems and risk of violence to the researcher. These individuals were identified during routine intake procedures carried out by custody officers with every detainee. This resulted in a small number of exclusions due to either intoxication and/or risk of violence to the researcher, but the exact number of exclusions was not specifically recorded. There were no exclusions due to serious communication/comprehension problems and/or serious mental health problems.

### Power calculation

In order to determine the sample size required to estimate the prevalence of ID and ADHD in this population with good precision we used the following formula provided by Daniel [42], and made a finite population correction following the recommendations of Naing and colleagues [43]:

$$\frac{n=Z^{2}P\left(1-P\right)}{d^{2}}$$

where n = sample size, Z = Z statistic for a level of confidence, *P* = expected prevalence or proportion (in proportion of one; if 20%, *P* = 0.2), and *d* = precision (in proportion of one; if 5%, *d* = 0.05).

As recommended, we used a standard 95% confidence level for both calculations for which the Z statistic is 1.96.

With regard to prevalence of ID, we based our calculation on an expected prevalence rate of 7.5% reported by Jackson [4] in probation services. This rate was selected because the study had used the Learning Disability Screening Questionnaire (LDSQ) (as used in the current study) and a population of offenders in the community rather than a secure setting. As the expected prevalence fell below 10%, Naing and colleagues [43] recommend that *d* should be half of *P*; therefore, the *d* value used for the ID calculation was 0.04 (4% expressed as a proportion of one). The estimated number of participants required was 164.

With regard to prevalence of ADHD, we based our calculation on an expected prevalence rate of 19% found in a recent UK study in probation services [44]. This rate was selected because it was obtained from a population of offenders in the community rather than a secure setting. The precision value of 5% was used for the ADHD calculation as the expected prevalence fell between the 10% and 90% band recommended; therefore, the *d* figure used was 0.05 (5% expressed as a proportion of one)”. The estimated number of participants required was 215.

The two estimated sample sizes are different mainly due to the different values of precision and expected prevalence used in the calculations, as described by Naing *et al*. [43].

### Measures

#### Barkley scales for ADHD in childhood and adulthood [45]

These self-report screens each comprise 18 items relating to Diagnostic and Statistical Manual of Mental Disorders IV (DSM-IV) classified symptoms of inattention, hyperactivity and impulsivity. On each screen, nine items relate to problems of hyperactivity/impulsivity and nine to inattention. Each item is rated on a four-point scale: ‘never or rarely’ (0), ‘sometimes’ (1), ‘often’ (2) and ‘very often’ (3). The individual is asked to rate items for their behavior in childhood and over the past six months (current/adulthood). The Barkley scales offer a total symptom count for the inattentive domain and the hyperactive/impulsive domain (0 to 9 for each) and the cut-off criteria applied were: (i) six or more ADHD symptoms in either domain for retrospective childhood ratings; plus (ii) four or more ADHD symptoms from either domain for ‘current’ ADHD ratings.

The Barkley scales also include an assessment of impairment asking participants to rate, on a four-point scale (0 = ‘never/rarely’, 1 = ‘sometimes’, 2 = ‘often’, and 3 = ‘very often’), the functional impact of endorsed ADHD symptoms. Impact is assessed for 10 domains, including home life, work or occupation, social interactions, community activities, educational activities, dating or marital relationships, money management, driving, leisure or recreational activities and management of daily responsibilities. The Barkley Current Symptom Scale has been reported to have 75% sensitivity and 61% specificity on the Inattention scale and 69% sensitivity and 39% specificity for the Hyperactivity/Impulsivity scale [46]. Little other psychometric data are available on this measure. However, it has the advantage of being based directly on the DSM-IV criteria for ADHD and is commonly used as a screening measure in research [45].

#### Diagnostic Interview for ADHD in Adults (DIVA) [47]

This semi-structured clinical interview was used to classify the diagnostic status of the sample. The interview systematically evaluates each of the 18 DSM-IV ADHD symptom items (9 each in the domains of attention and hyperactivity/impulsivity) for both current (that is, in the past six months) and childhood symptoms. The DIVA also includes additional questions to establish impairment from ADHD symptoms (impairment criteria), in two or more settings (pervasiveness criteria) and the age of onset of symptoms (age of onset criteria). The DSM-IV recommendation is that, for a positive diagnosis, six items must be endorsed in the same (or both) domain(s) for current and childhood symptoms. However, a distinction is made in the literature between ‘syndromatic’ and ‘symptomatic’ ADHD, with the latter requiring endorsement of fewer current symptoms (four, rather than six, out of nine) [48]. This identifies those people in partial remission of symptoms, and was applied in the current study in order to reduce the likelihood of a Type II error at the screening stage.

#### Learning Disability Screening Questionnaire (LDSQ) [15,49]

This questionnaire rates an individual based on seven items tapping intellectual skills (for example, can the person read and write) and functional skills (for example, can the person live independently). A higher score (range 0 to 7) indicates lower likelihood of intellectual disability and scores can be converted to a percentage score to be compared with the cut-off percentage, as described in the manual. The LDSQ has been reported to have good convergent validity when compared with full-scale IQ scores [15,50], with over 80% specificity and sensitivity, and has been validated for use in forensic settings where it is also found to have acceptable psychometric properties [15].

#### Oregon Adolescent Depression Project - Conduct Disorder Screen (OADP-CDS) [51]

This six-item self-report screen asks individuals to rate frequency of adolescent conduct behaviors on a four-point scale. Possible scores range from 6 to 24 with a higher score indicating greater presence of conduct disorder symptoms. The OADP-CDS has been shown to have good internal consistency, test-re-test reliability and good screening efficiency for detecting lifetime CD. As recommended, cut-off scores of 10 or higher for males and 8 or higher for females were used to indicate presence of CD [51]. In the present study, CD was measured retrospectively with an older age group and this methodology has been used successfully previously in an older sample [25].

#### Custody Record review

A review of the Custody Record was conducted in order to extract relevant information to test the hypotheses of the present study. Data were collected relating to: (1) police procedures and provisions (that is, if interviewed by police, seen by a HCP, Appropriate Adult required). (2) duration of time the detainee had spent in custody (that is, time elapsed between the individual being detained and the time of their record review being completed by the researchers). (3) requests made while in custody; this was the number of times each participant requested food/drink, legal contact (for example, solicitor), family contact, or made some other request (for example, for a blanket). Information pertaining to these requests was found to be recorded routinely using similar wording across the small number of staff responsible for responding to a buzzer pressed by a detainee. There were no record reviews completed where this information was not available.

#### Custody Record risk assessment screening tool

This tool is a standard assessment that is routinely administered to all detainees presenting at the custody suite reception. It is documented within the Custody Record. The tool consists of categorical data in the form of Yes/No answers for questions pertaining to alcohol and substance use, medical and/or mental health needs, ability to read and write, and additional needs while in custody. Four questions completed by the Custody Officer and seven of the detainee-report questions were thought to be theoretically relevant to exploring whether the screening tool successfully identified vulnerable individuals.

### Procedure

Following ethical approval by the King’s College London Psychiatry, Nursing and Midwifery subcommittee (reference PNM/10/11-116), participants were recruited at the custody suite over a 12-week period in 2011. All detainees deemed suitable by custody staff were approached by a researcher with a member of staff. They were invited to take part in a study investigating factors that might be associated with behavior in custody. Those who were interested in participating were taken to a consultation room off the main custody area where the research assistant provided an information sheet and a verbal explanation of the study, and the participant had the opportunity to ask any questions. Those who reported difficulties reading the information sheet, or those who did not wish to do so, had the information sheet verbally read to them with opportunities to ask questions and clarify details as required. No individuals reported specific problems understanding the material when it was verbally presented to them. Participants who then wished to take part provided their written consent and completed the questionnaires. Around one-third of participants completed the questionnaires verbally with the researcher due to self-reported difficulties with reading. Following the completion of the participant measures, the researcher completed the record review. Ten participants consented to complete questionnaires but did not give consent for researchers to examine their Custody Record. With regard to the questionnaires, there was some missing data due to some detainees not wishing to complete some of the measures. The precise number completed on each screening measure is reported in the Results section.

## Results

### Prevalence rates in custody

#### Intellectual disability

Of the 195 participants who completed the LDSQ, 13 (6.7%) screened positive for intellectual disability (see Table 1).

**Table 1:** Diagnostic classifications based on the screening scales

**Diagnosis**	**n (%)**
**Intellectual disability (n = 195)**	
No intellectual disability	182 (93.3)
Intellectual disability	13 (6.7)
**Childhood ADHD (n = 196)**	
No ADHD	133 (67.9)
ADHD	63 (32.1)
*Combined*	*42 (21.4)*
*Inattentive*	*11 (5.6)*
*Hyperactive/impulsive*	*10 (5.1)*
**Current ADHD (n = 196)**	
No ADHD	150 (76.5)
ADHD	46 (23.5)
*Combined*	*29 (14.8)*
*Inattentive*	*4 (2.0)*
*Hyperactive/impulsive*	*13 (6.6)*
**Childhood conduct disorder (n = 194)**	
No conduct disorder	48 (23.7)
Conduct disorder	148 (76.3)

### Attention deficit hyperactivity disorder

Four participants did not wish to complete the childhood screen. Of the remaining 196, 63 (32.1%) screened positive for ADHD in childhood and 46 (23.5%) for current symptoms with the largest subgroups being combined (14.8%) and hyperactive/impulsive (6.6%) (see Table 1). All 46 participants were invited to complete the DIVA and 24 did so (9 did not wish to complete the interview and withheld consent for this part of the study, 11 interviews were interrupted due to pressure on room space and/or time, and 2 individuals withdrew participation before completing the interview). A further four participants stopped half way through the DIVA (as they were released from custody), although they met criteria for predominantly inattentive type (this being the only section of the interview they had completed by that time). From the 24 completed interviews, 19 participants (79.2%) had persisting ADHD symptoms (that is, symptomatic persistence) and 14 individuals (58.3%) had syndromatic persistence (that is, met full DSM-IV criteria). Hence, sensitivity of the screen was estimated to be 79%, which when applied adjusted the prevalence rate for current ADHD down from 23.5% to a more conservative estimate of 18.5%.

A period of civil unrest occurred in London during the time of the study known as the ‘London riots’. Screening rates of childhood ADHD in participants processed in the two weeks following the London riots increased from 32% to 38%. Rates of current symptoms remained consistent; 23.5% and 24%, respectively. However, the increase in the child rates was not significant.

### Conduct disorder

Of the 194 participants who completed the OADP-CDS, 148 (76.3%) screened positive for childhood CD (see Table 1).

### Co-morbidity

Of those who screened positive for ADHD (n = 46), 44 (95.7%) completed the CD screen, all of whom screened positive for CD, and 6 (13%) had co-morbid ID. Table 2 shows correlations (two-tailed) between continuous scores on the diagnostic screens, including the Barkley impairment score, all of which were significant. We used Cohen’s recommendation for correlations of .10, .30 and .50 as indicators of small, medium and large effect sizes, respectively [52]. ADHD childhood and current symptoms were highly correlated with CD (large effect size), and more modestly correlated with ID (medium effect size). CD was most strongly correlated with childhood (r = .77) and current (r = .65) ADHD symptoms, explaining 54% and 43% of the variance, respectively.

**Table 2:** Correlations between total scores on the diagnostic screens

	**Mean (SD) (N)**	**Barkley ADHD adult total**	**Barkley current impairment score**	**LDSQ percentage score**	**Oregon CD total**
Barkley ADHD child total	21.9 (14.0) (196)	0.72**	.55**	-.35**	.77**
Barkley ADHD adult total	17.3 (12.2) (196)	-	.79**	-.34**	.65**
Barkley current impairment score	8.2 (7.4) (195)	-	-	-.23*	.51**
LDSQ percentage score	78.5 (18.9) (195)	-	-	-	-.30**
Oregon CD total	13.3 (4.9) (194)				

*P < .01.

**P < .001.

ADHD, Attention Deficit Hyperactivity Disorder; CD, Conduct disorder; LDSQ, Learning Disability Screening Questionnaire.

Table 2 shows that there was a significant correlation between CD and ID (r = −.30; medium effect size), but this became non-significant (r = −.11; small effect size) after controlling for current (adult) ADHD symptoms in a partial correlation.

### Requests made on police time

In order to investigate the relationship of CD, ADHD and ID with requests made for staff time while in custody, one-tailed partial correlations were conducted between symptoms (continuous screening scores) and the number of requests made to staff while in custody. The duration of time between the individual being detained and the time of their record review was recorded and controlled for, as longer time would have afforded greater opportunity to make requests (r = .62, *P* < .001). Results indicated significant positive correlations between the number of requests made and childhood CD (r = .23, *P* < .01), childhood ADHD (r = .23, *P* < .01), current ADHD symptoms (r = .39, *P* < .001), ADHD impairment (r = .29, *P* < .001), but not for ID (r = −.07, ns).

In order to explore if detainees who were currently symptomatic for ADHD would consume staff resources over and above CD, a hierarchical multiple regression was conducted (see Table 3). CD (Oregon total score) was entered in the first block along with time spent in custody prior to the psychological assessment. ADHD current symptoms were added in Block 2. In Block 1, CD (β = .19) and length of time in custody (β = .60) contributed significantly to the variance in requests for staff time, explaining 41% of the variance in the number of requests made. Entering ADHD symptoms in Block 2 added 6% to the total variance (47%), with only ADHD (β = .31) and time spent in custody (β = .59) contributing significantly to the model, thus showing that adding ADHD symptoms eliminated the effect of CD.

**Table 3:** Summary of multiple regression analysis (hierarchical) for CD and current ADHD for predicting requests

		**B**	**Standard error B**	**β**	**t**	**Adjusted R**^**2**^
Block 1	Constant	−3.26	1.27		−2.6*	.41 F(2,177) = 63.5**
	Conduct disorder	.28	.09	.19	3.2*	
	Time in custody	.28	.03	.60	10.5**	
Block 2	Constant	−2.39	1.23		−1.96	
	Conduct disorder	-.02	.01	-.01	-.16	
	Time in custody	.27	.02	.59	10.9**	
	Current ADHD symptoms	.18	.04	.31	4.5**	.47 F(3,176) = 53.5**

*P < .05.

**P < .001.

ADHD, Attention Deficit Hyperactivity Disorder; CD, Conduct Disorder.

### Identification of vulnerabilities using the custody risk assessment procedures

Chi-square analyses were conducted to investigate the likelihood of individuals screening positively for ADHD endorsing particular items on the Custody Record risk assessment screening tool. The screening classifications were used rather than the diagnostic interview for ADHD as this would have substantially decreased the number of participants included in the analysis. The results are provided in Table 4. Those screening positive for ADHD were significantly more likely to have endorsed items on the self-report risk assessment referring to having an existing illness or injury or other medical condition (OR = 2.27), substance use (OR = 3.42), history of self-harm (OR = 3.57), or current mental health problems (OR = 4.37). On the custody officer report, the only item that was significant was indications of self-harm (OR = 3.64).

**Table 4:** Differences between those screening positive or negative for ADHD in endorsement of risk assessment items

**Self-report risk assessment**	***Positive ADHD screen % (n)***	***Negative ADHD screen % (n)***	***Χ***^***2 ***^ ***(df = 1)***	***Odds ratio (95% confidence interval)***
Any illness or injury, or any other medical condition?	51.3 (20)	31.7 (45)	5.10*	2.27 (1.10 to 4.66)
Taking, supposed to be taking, or need any tablets/medication?	19.5 (8)	15.6 (22)	0.35	1.31 (0.54 to 3.21)
Consumed alcohol in the last 24 hours?	28.2 (11)	26.8 (38)	0.03	1.08 (0.49 to 2.37)
Dependent on drugs or any other substances?	25.6 (10)	9.2 (13)	7.50**	3.42 (1.37 to 8.57)
Ever tried to harm yourself?	23.1 (9)	7.7 (11)	7.32**	3.57 (1.36 to 9.39)
Experiencing any mental health problems or depression?	23.1 (9)	6.4 (9)	9.35**	4.37 (1.60 to 11.94)
Require any help with reading/writing?	10.3 (4)	2.8 (4)	3.96	3.91 (0.93 to 16.44)
Custody officer risk assessment				
Detainee appears to be injured or unwell?	20.5 (8)	11.3 (16)	2.22	2.02 (0.79 to 5.14)
Detainee is in need of a Doctor or other Health Care Professional?	20.0 (6)	11.3 (13)	1.58	1.96 (0.68 to 5.69)
Appears to have taken/be under the influence of alcohol/drugs/any other substance?	23.1 (9)	12.8 (18)	2.55	2.05 (0.84 to 5.01)
Detainee has indications of self harm?	17.9 (7)	5.7 (8)	6.03*	3.64 (1.23 to 10.77)

*P < .05.

**P < .01.

ADHD, Attention Deficit Hyperactivity Disorder.

A parallel analysis was not performed for the ID group in view of the very small sample who were likely to meet the criteria for ID.

Out of the 190 individuals who had consented for their Custody Record to be viewed, 85 (44.7%) were noted to have been seen by a HCP. Of these individuals, the current study identified that 7 people screened positive for ID and 24 screened positive for current ADHD. However, only two (28.6%) and one (4.2%) had an Appropriate Adult, respectively.

Out of 166 individuals interviewed by police while in custody, 7 (4.2%) were provided with an Appropriate Adult. Two of these suspects screened positive for ID and one for ADHD. Of the remaining 159, 35 (22.0%) screened positive for current ADHD and 9 (5.7%) screened positive for ID.

## Discussion

The findings in this study mostly support our hypotheses. The prevalence rates of ID and ADHD were both higher than those reported in the general population from normative (general population) data. With regard to ADHD, rates were marginally higher for childhood symptoms during a period of civil unrest, which suggests that rates of ADHD in custody may be influenced by local and situational factors for young detainees. As expected, there were high rates of co-morbidity, especially between ADHD and CD, which are known to occur together at a higher rate than chance [39]. We found the shared variance of CD with childhood and current ADHD symptoms to be 59% and 44%, respectively, and CD was associated with ID because of its comorbidity with ADHD.

Our results indicate that ADHD and CD symptoms predicted consumption of staff time, with ADHD symptoms driving requests of staff time after controlling for CD and duration of time spent in custody. One possible explanation for this is that those who are symptomatic for ADHD have increased behavioral disinhibition (for example, [28]) where impatience and restlessness may be expressed as requests being made of staff. It is not possible to comment on the legitimacy of the requests made or the time taken to deal with each request. This would be important to determine in future research as the apparent increased demand of people with ADHD in restricted custodial settings has staffing and resource implications. ID was not significantly correlated with requests for staff time, which may have been due to the fact that very few of the detainees performed poorly on the LDSQ. This supports the findings of Gudjonsson and colleagues from the Royal Commission Study [2,3] and the view of Murphy and Mason [6] that very few people with severe ID are likely to have contact with the CJS. An alternative explanation for the non-significant relationship is that individuals with intellectual disability may be particularly passive (for example, [53]) and not make requests of staff.

The exploratory analysis found that the implementation of improved assessment procedures had not increased the rate of the use of Appropriate Adults beyond that reported 20 years previously. Indeed, only two of the detainees screening positive for ID and one for ADHD had been provided with an Appropriate Adult. This represents a serious flaw in the current risk assessment process, especially given the recent initiative of HCPs being present at police custody suites.

The indicated rate of ID in the current study (6.7%) is consistent with previous findings [2] and suggests an increase from that found in the general population (for example, [54]). The rate is substantially lower than that reported among offenders in the Søndenaa *et al*. study [55], but it is consistent with that reported in the Bradley Report [1]. Research has shown that many people who have had their conviction overturned on appeal are of low intelligence [7,13,56-58]. Therefore, early identification of their vulnerabilities may prevent wrongful convictions [9]. The same holds true for people with ADHD. They are even less readily diagnosed than those with ID, in spite of the condition being more common, and the vulnerabilities associated with their condition are not so well established within the CJS [59]. In the current study, the detainees with ADHD showed almost a 10-fold increase from the adult general population when screens were used and about a 7-fold increase when a diagnostic interview was used. We found that the rate of ADHD was three to four times higher than that for ID, which suggests that there are going to be many more detainees at police stations with ADHD than ID. Therefore, appropriate screening for people with ADHD should in future be incorporated into the routine screening and HCP assessment and the findings appropriately used to inform a decision on the need for an Appropriate Adult.

Although every individual brought into custody completed a risk-assessment (completed by themselves and by a Custody Officer), and almost half (44.7%) of this sample were interviewed by a HCP, very few were identified as having difficulties that merited the need for an Appropriate Adult. This under-identification has been recognized as one of the main barriers to providing adequate support (for example, [15]). Nevertheless, it is positive that items of the current risk assessment tool show promise for the identification of self-reported mental health problems and officer-reported self-harming behaviors in people with ADHD. This supports the work of Clare and Gudjonsson [3], highlighting the importance of self-reported vulnerabilities by persons detained at police stations as a potential ‘red flag’ for an Appropriate Adult.

Disappointingly, of those detainees interviewed by police only 4.2% were provided with an Appropriate Adult and this figure is almost identical to the 4.3% reported over 20 years previously in the Royal Commission study [2]. This shows that the current risk assessment practices are failing and/or are not influencing the behavior of the police in terms of required provision of Appropriate Adults. Some of the risk assessment indicators, such as serious reading and writing problems, should have alerted the police and the HCP staff to the need for an Appropriate Adult. This suggests that the HCP staff employed at the police station do not focus sufficiently on psychological or mental health symptoms, including those associated with ADHD and ID. These individuals require an Appropriate Adult during police interviews and other formal procedures (for example, reading and signing documents), which they do not receive. This practice needs to change.

The findings should be considered in light of some particular strengths and limitations of the study. Although only representing one large Metropolitan Police station, the findings are likely to generalize to other police stations [2], but some regional variations may exist for the custodial and interview process [13]. The study also has merit in the large sample of detainees who participated, and our power calculations indicated that the study was well-powered to screen for ID but marginally underpowered to screen for ADHD. Nevertheless, this is the first study of ADHD prevalence and associated behavior in police custody that has included a full clinical diagnostic methodology to estimate ADHD prevalence. The researchers were not qualified healthcare practitioners in diagnosing ADHD. However, they were trained to a level of good reliability by qualified clinical practitioners with expertise in the assessment of ADHD in adulthood and using the DIVA semi-structured interview.

Other than the prevalence data for ADHD using a clinical diagnostic methodology, all other analyses were conducted based on a sample obtained from screening rates of ADHD, CD and ID. Hence, diagnostic rates cannot be estimated for CD and ID as the number of false positive and false negative identifications are unknown. In clinical practice, for example, a diagnosis of ID would require assessment of both cognitive and adaptive functioning, and an assessment of childhood onset. Secondly, the screening and diagnostic data were based on self-reported information. This was a necessary methodological limitation as the high turnover of detainees in the custody suite and the short periods of time they generally spend in custody limited the opportunity for obtaining supplementary informant information. Nevertheless, adults with ADHD have been found to be reliable in reporting attention problems [60]. There was also no detailed clinical interview of current symptoms relevant to mental illness, such as depression. Gudjonsson *et al*. [2] found that agitation and depressive symptoms were commonly found in a police station sample. Furthermore, anxiety and depression among suspects detained for interviews have been associated with the reporting of false confessions [61].

Finally, the focus of the current study has been on identifying vulnerabilities considered relevant to police custody procedures in relation to safeguards and reliability of police interviews. However, a further benefit of thorough screening procedures would be to signpost individuals who may require more detailed assessments for support within designated external services.

## Conclusion

In summary, ID and ADHD are recognized vulnerabilities for navigating the criminal justice system, particularly early on during interviews and court proceedings. As predicted, these difficulties are more common in a police custody setting than in the general population, yet are largely unrecognized. Identification of these potential vulnerabilities does not appear to have improved greatly with the introduction of the current risk-assessment tools. The self-report version provided a more accurate predictor in the current study than the Custody Officers’ evaluation, although this seems to be disregarded as it does not appear to be routinely used to inform needs in custody. In turn, this resulted in a substantial number of individuals who self-reported literacy and/or mental health problems not being provided with the supportive mechanisms that they may be entitled to during the police interview process. What is clear is that Custody Officers and HPC staff need to develop greater awareness about the mental health and/or intellectual vulnerabilities presenting in police detainees, including their screening and management. This knowledge and information is essential to safeguard the police interview process through the implementation of effective systems to determine the provision of Appropriate Adults.

## Abbreviations

ADHD: Attention Deficit Hyperactivity Disorder; CD: Conduct disorder; CJS: Criminal Justice System; DIVA: Diagnostic Interview for ADHD in Adults; DSM-IV: Diagnostic and Statistical Manual of Mental Disorders-IV; HCPs: Healthcare practitioners; ID: Intellectual disabilities; LDSQ: Learning Disability Screening Questionnaire; OADP-CDS: Oregon Adolescent Depression Project - Conduct Disorder Screen; PACE: Police and Criminal Evidence Act.

## Competing interests

SY has been a consultant for Janssen-Cilag, Eli-Lilly and Shire; has given educational talks at meetings sponsored by Janssen-Cilag, Shire and Flynn-Pharma, Novatis, Eli-Lilly; and has received research grants from the National Institute of Health Research, Janssen-Cilag, Eli-Lilly and Shire. SY was a member of the NICE guideline development group for ADHD. GG has received speakers’ fees and travel honoraria from Janssen, Eli-Lilly and Shire. EG and OS have no disclosures.

## Authors’ contributions

All authors have contributed to data analysis and producing drafts of this article through electronic discussions and face-to-face meetings. The final manuscript was read and approved by all authors. EG and OS were the Research Assistants who conducted the data collection.

## Pre-publication history

The pre-publication history for this paper can be accessed here:

http://www.biomedcentral.com/1741-7015/11/248/prepub

## References

[B1] Bradley K The Bradley Report: Lord Bradley’s Review of People with Mental Health Problems or Learning Disabilities in the Criminal Justice System 2009 London: Department Health [http://webarchive.nationalarchives.gov.uk/20130107105354/http://www.dh.gov.uk/prod_consum_dh/groups/dh_digitalassets/documents/digitalasset/dh_098698.pdf]

[B2] Gudjonsson G Clare ICH Rutter S Pearse J Persons at Risk during Interviews in Police Custody: The Identification of Vulnerabilities. Royal Commission on Criminal Justice Report. Cmnd.2263 1993 London: HMSO

[B3] Clare ICH Gudjonsson GH Devising and Piloting a New “Notice to Detained Persons”. Royal Commission on Criminal Justice 1992 London: HMSO

[B4] Jackson M Learning Disabilities - Wakefield Probation Service - Final Report 2011 London: Department of Health

[B5] Holland T Clare IC Mukhopadhyay T Prevalence of ‘criminal offending’ by men and women with intellectual disability and the characteristics of ‘offenders’: implications for research and service development J Intellect Disabil Res 2002 11 6 20 12061335 10.1046/j.1365-2788.2002.00001.x

[B6] Murphy GH Mason J Bouras N, Holt G People with intellectual disabilities who are at risk of offending Psychiatric and Behavioural Disorders in Intellectual and Developmental Disabilities 2007 Cambridge, UK: Cambridge University Press 173 201

[B7] Gudjonsson GH Invited article. Psychological vulnerabilities during police interviews. Why are they important? Leg Criminol Psychol 2010 11 161 175 10.1348/135532510X500064

[B8] Gudjonsson GH False confessions and correcting injustices N Engl Law Rev 2012 11 689 709

[B9] Gudjonsson GH Grisso T Felthous AR, Sass H Legal competencies in relation to confession evidence International Handbook on Psychopathic Disorders and the Law, Volume 2 2008 Chichester, UK: John Wiley & Sons 177 187

[B10] Gudjonsson GH Joyce T Interviewing adults with intellectual disabilities Adv Ment Health Intellect Disabil 2011 11 16 17 10.5042/amhid.2011.0108

[B11] Gudjonsson GH Young S Bramham J Interrogative suggestibility in adults diagnosed with attention-deficit hyperactivity disorder (ADHD), a potential vulnerability during police questioning Personal Individ Differ 2007 11 737 745 10.1016/j.paid.2007.01.014

[B12] Office H Police and Criminal Evidence Act 1984 1985 London: HMSO

[B13] Gudjonsson GH The Psychology of Interrogations and Confessions. A Handbook 2003 Chichester, UK: John Wiley & Sons

[B14] Medford S Gudjonsson G Pearse J The Identification of Persons at Risk in Police Custody. The Use of Appropriate Adults by the Metropolitan Police 2000 London: Jointly published by the Institute of Psychiatry and New Scotland Yard

[B15] McKenzie K Michie A Murray A Hales C Screening for offenders with an intellectual disability: the validity of the Learning Disability Screening Questionnaire Res Dev Disabil 2012 11 791 795 10.1016/j.ridd.2011.12.006 22245728

[B16] Home Office: Police and Criminal Evidence Act 1984 (PACE) Code C. Code of Practice for the Detention, Treatment and Questioning of Persons by Police Officers 2012 London: HMSO

[B17] American Psychiatric Association Diagnostic and Statistical Manual of Mental Disorders. DSM-IV 1994 Washington, DC: American Psychiatric Association

[B18] Bramham J Ambery F Young S Morris R Russell A Xenitidis K Asherson P Murphy D Executive functioning differences between adults with Attention Deficit Hyperactivity Disorder and autistic spectrum disorder in initiation, planning and strategy formation Autism 2009 11 245 264 10.1177/1362361309103790 19369387

[B19] Rose ED Bramham J Young SJ Paliokosta E Xenitidis K Neuropsychological characteristics of adults with comorbid ADHD and borderline/mild intellectual disability Res Dev Disabil 2009 11 496 502 10.1016/j.ridd.2008.07.009 18774690

[B20] Young S Morris RG Toone BK Tyson C Planning ability in adults diagnosed with Attention-Deficit/Hyperactivity Disorder: a deficit in planning ability Neuropsychology 2007 11 581 589 17784806 10.1037/0894-4105.21.5.581

[B21] Young S Gudjonsson G Growing out of Attention-Deficit/Hyperactivity Disorder: the relationship between functioning and symptoms J Atten Disord 2008 11 162 169 17494827 10.1177/1087054707299598

[B22] Gudjonsson GH Wells J Young S Personality disorders and clinical syndromes in ADHD prisoners J Atten Disord 2012 11 305 314 10.1177/1087054710385068 20978272

[B23] Adamou M Arif M Asherson P Bolea B Coghill D Gudjonsson G Halmøy A Hodgkins P Müller U Pitts M Trakoli A Williams N Young S Occupational issues of adults with ADHD BMC Psychiatry 2013 11 59 10.1186/1471-244X-13-59 23414364 PMC3599848

[B24] Gudjonsson GH Sigurdsson JF Einarsson E Bragason OO Newton AK Interrogative suggestibility, compliance and false confessions among prisoners and their relationship with attention deficit hyperactivity disorder (ADHD) symptoms Psychol Med 2008 11 1037 1044 18275632 10.1017/S0033291708002882

[B25] Gudjonsson G Sigurdsson JF Sigfusdottir ID Young SA A national epidemiological study of offending and its relationship with ADHD symptoms and associated risk factors J Atten Disord 2012 [Epub ahead of print] 10.1177/1087054712437584 22522573

[B26] Young S Gudjonsson GH Wells J Asherson P Theobald D Oliver B Scott C Mooney A Attention Deficit Hyperactivity Disorder and critical incidents in a Scottish prison population Pers Indiv Differ 2009 11 265 269 10.1016/j.paid.2008.10.003

[B27] Young S Misch P Collins P Gudjonsson GH Predictors of institutional behavioural disturbance and offending in the community among young offenders J Forensic Psychiatry Psychol 2011 11 72 86 10.1080/14789949.2010.495991

[B28] Gudjonsson GH Sigurdsson JF Adalssteinsson T Young S The relationship between ADHD symptoms, mood instability, and self-reported offending J Atten Disord 2013 11 339 346 10.1177/1087054711429791 22290695

[B29] Willcutt EG The prevalence of DSM-IV attention-deficit/hyperactivity disorder: a meta-analytic review Neurotherapeutics 2012 11 490 499 10.1007/s13311-012-0135-8 22976615 PMC3441936

[B30] Simon V Czobor P Balint S Meszaros A Bitter I Prevalence and correlates of adult attention-deficit hyperactivity disorder: meta-analysis Br J Psychiatry 2009 11 204 211 10.1192/bjp.bp.107.048827 19252145

[B31] Gudjonsson GH Sigurdsson JF Young S Newton AK Peersen M Attention Deficit Hyperactivity Disorder: how do ADHD symptoms relate to personality among prisoners? Personal Individ Differ 2009 11 64 68 10.1016/j.paid.2009.01.048

[B32] Rösler M Retz W Retz-Junginger P Hengesch G Schneider M Supprian T Schwitzgebel P Pinhard K Dovi-Akue N Wender P Thome J Prevalence of attention deficit-/hyperactivity disorder (ADHD) and comorbid disorders in young male prison inmates Eur Arch Psychiatry Clin Neurosci 2004 11 365 371 10.1007/s00406-004-0516-z 15538605

[B33] Young S Gudjonsson GH Misch P Collins P Carter P Redfern J Goodwin E Prevalence of ADHD symptoms among youth in a secure facility: the consistency and accuracy of self- and informant-report ratings J Forensic Psychiatry Psychol 2010 11 238 246 10.1080/14789940903311566

[B34] Rasmussen K Almvik MR Levander S Attention Deficit Hyperactivity Disorder, reading disability, and personality disorders in a prison population J Am Acad Psychiatry Law 2001 11 186 193 11471785

[B35] Ginsberg Y Hirvikoski T Lindefors N Attention Deficit Hyperactivity Disorder (ADHD) among longer-term prison inmates is a prevalent, persistent and disabling disorder BMC Psychiatry 2010 11 112 10.1186/1471-244X-10-112 21176203 PMC3016316

[B36] Satterfield JH Faller KJ Crinella FM Schell AM Swanson J Homer LD A 30-year prospective follow-up study of hyperactive boys with conduct problems: adult criminality J Am Acad Child Adolesc Psychiatry 2007 11 601 610 10.1097/chi.0b013e318033ff59 17450051

[B37] Mordre M Groholt B Kjelsberg E Sandstad B Myhre AM The impact of ADHD and conduct disorder in childhood on adult delinquency: a 30 years follow-up study using official crime records BMC Psychiatry 2011 11 57 10.1186/1471-244X-11-57 21481227 PMC3082292

[B38] Gunter TD Arndt S Riggins-Caspers K Wenman G Cadoret RJ Adult outcomes of attention deficit hyperactivity disorder and conduct disorder: are the risks independent or additive? Ann Clin Psychiatry 2006 11 233 237 10.1080/10401230600948415 17162622

[B39] Waschbusch DA A meta-analytic examination of comorbid Hyperactive Impulsive-Attention problems and conduct problems Psychol Bull 2002 11 118 150 11843545 10.1037/0033-2909.128.1.118

[B40] Ahuja A Martin J Langley K Thapar A Intellectual disability in children with Attention Deficit Hyperactivity Disorder J Pediatr 2013 11 890 895 10.1016/j.jpeds.2013.02.043 23608559 PMC4078221

[B41] National Policing Improvement Agency Guidance on the Safer Detention and Handling of Persons in Police Custody 2012 2 London: National Policing Improvement Agency

[B42] Daniel WW Biostatistics: A Foundation for Analysis in the Health Sciences 1999 New York: John Wiley & Sons

[B43] Naing L Winn T Rusli BN Practical issues in calculating the sample size for prevalence studies Arch Orofac Sci 2006 11 9 14

[B44] Adamou M Prevalence of Attention Deficit Hyperactivity Disorder in the West Yorkshire probation population Presented at the ‘From Classroom to Courtroom: ADHD and the Criminal Justice System’ Conference 2012 Crowthorne, U K: Broadmoor Hospital

[B45] Barkley RA Attention Deficit/Hyperactivity Disorder: A Handbook for Diagnosis and Treatment 1998 2 New York: Guilford Press

[B46] Quinn CA Detection of malingering in assessment of adult ADHD Arch Clin Neuropsychol 2003 11 379 395 14591453

[B47] Kooij JJ Francken MH Diagnostic Interview for ADHD in Adults 2010 The Hague, Netherlands: Diva Foundation Available from http://www.psyq.nl/files/1263005/DIVA_2_EN.pdf

[B48] Kooij JJ Buitelaar JK van den Oord EJ Furer JW Rijnders CA Hodiamont PP Internal and external validity of attention-deficit hyperactivity disorder in a population based sample of adults Psychol Med 2005 11 817 827 10.1017/S003329170400337X 15997602

[B49] McKenzie K Paxton D Learning Disability Screening Questionnaire 2005 Edinburgh, UK: GCM Records

[B50] McKenzie K Paxton D Promoting access to services: the development of a new screening tool Learn Disabil Pract 2006 11 17 21 10.7748/ldp2006.07.9.6.17.c7451

[B51] Lewinsohn PM Rohde P Farrington DP The OADP-CDS: a brief screener for adolescent conduct disorder J Am Acad Child Adolesc Psychiatry 2000 11 888 895 10.1097/00004583-200007000-00018 10892231

[B52] Cohen J A power primer Psychol Bull 1992 11 155 159 19565683 10.1037//0033-2909.112.1.155

[B53] Knox M Mok M Parmenter TR Working with the experts: collaborative research with people with an intellectual disability Disabil Soc 2000 11 49 61 10.1080/09687590025766

[B54] Emerson E Hatton C Robertson J Roberts H Baines S Evison F Glover G People with Learning Disabilities in England 2011 2012 Durham: Improving Health and Lives: Learning Disability Public Health Observatory

[B55] Søndenaa E Rasmussen K Nøttestad JA Forensic issues in intellectual disability Curr Opin Psychiatry 2008 11 449 453 10.1097/YCO.0b013e328305e5e9 18650685

[B56] Drizin SA Leo RA The problem of false confessions in the post-DNA world N C Law Rev 2004 11 891 1007

[B57] Garrat BL Convicting the Innocent 2011 London: Harvard University Press

[B58] Perske R False confessions from 53 persons with intellectual disabilities: the list keeps growing Intellect Dev Disabil 2008 11 468 479 10.1352/2008.46:468-479 19006433

[B59] Young S Adamou M Bolea B Gudjonsson G Müller U Pitts M Thome J Asherson P The identification and management of ADHD offenders within the criminal justice system: a consensus statement from the UK Adult ADHD Network and criminal justice agencies BMC Psychiatry 2011 11 116 10.1186/1471-244X-11-116 21332994 PMC3050801

[B60] Young S Gudjonsson GH Neuropsychological correlates of the YAQ-S and YAQ-I self- and informant-reported ADHD symptomatology, emotional and social problems and delinquent behaviour Br J Clin Psychol 2005 11 47 57 10.1348/014466504X197769 15826343

[B61] Sigurdsson JF Gudjonsson GH Einarsson E Gudjonsson G Differences in personality and mental state between suspects and witnesses immediately after being interviewed by the police Psychol Crime Law 2006 11 619 628 10.1080/10683160500337808

